# Effect of Wendan decoction granules on preventing endolymphatic hydrops and protecting vestibular function in Guinea pigs

**DOI:** 10.1186/s41065-025-00516-2

**Published:** 2025-07-26

**Authors:** Jingjing Xia, Tiancheng Xie, Jinli Liu, Weikang Cheng, Longyan Liao, Ming Chen, Haibing Hua, Xiaobing Hou

**Affiliations:** 1 Department of Otolaryngology, Jiangyin Hospital Affiliated to Nanjing, University of Chinese Medicine, Jiangyin, 214413 Jiangsu China; 2 School of Clinical Medicine, Chengdu University of Traditional Chinese Medicine, Chengdu, 611137 China; 3 Department of Endocrinology, Jiangyin Hospital Affiliated to Nanjing, University of Chinese Medicine, Jiangyin, 214413 Jiangsu China; 4 Department of Gastroenterology, Jiangyin Hospital Affiliated to Nanjing, University of Chinese Medicine, Jiangyin, 214413 Jiangsu China

**Keywords:** Characterization of the chemical substance base, Endolymphatic hydrops, Vestibular system, Wendan decoction

## Abstract

**Objective:**

The aim of this study is to assess the therapeutic effects of Wendan decoction on endolymphatic hydrops (EH), a condition characterized by excessive inner ear fluid accumulation that is associated with Meniere’s disease, in guinea pigs, offering insights for clinical application.

**Methods:**

The active components and action targets of compound Wendan decoction were identified using BATMAN-TCM in this study. Gene Ontology (GO) analysis and Kyoto Encyclopedia of Genes and Genomes (KEGG) pathway enrichment analysis were conducted on Metascape and bioinformatics.com.cn platforms. Cytoscape was used to construct the TCM-compound-target-disease network and the disease-target pathway network for visual representation. The chemical profile of Wendan decoction was characterized through UHPLC-QTOF/MS. Guinea pigs were used to establish an EH model. The effects of Wendan decoction granules on neurological function, cochlear hydrops, the ultrastructure of the cochlear Corti organ area, inflammatory response, and the expression of AQP2 and P38MAPK in guinea pigs were examined using behavioral experiments, hematoxylin and eosin staining, scanning electron microscopy, ELISA, qRT-PCR, and immunohistochemistry staining.

**Results:**

A total of 103 components, primarily flavonoids and triterpenoid saponins, were identified in Wendan decoction. Wendan decoction significantly inhibited the MAPK signaling pathway. Wendan decoction granules alleviated neurological impairment induced by EH. Following treatment, guinea pigs exhibited reduced membranous labyrinth hydrops and decreased outer hair cell loss. Wendan decoction granules also suppressed the release of pro-inflammatory factors and reduced AQP2 protein expression in model guinea pigs.

**Conclusion:**

Wendan decoction granules effectively alleviated neurological impairment and inflammatory responses, preserved cochlear structure, reduced inflammation, and modulated AQP2 expression, offering a strong basis for further investigation of its therapeutic potential.

**Supplementary Information:**

The online version contains supplementary material available at 10.1186/s41065-025-00516-2.

## Graphical Abstract

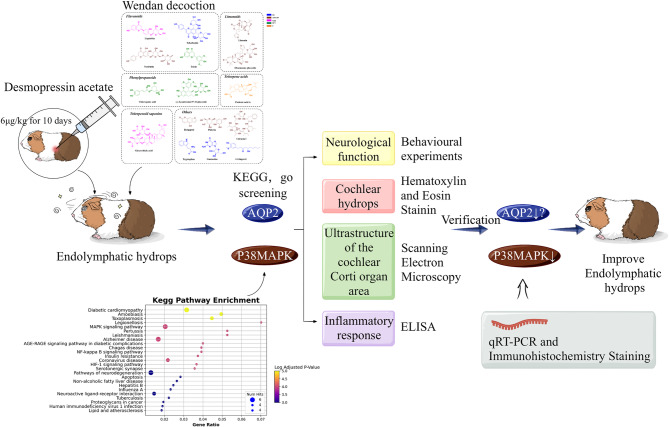

## Introduction

The inner ear is located in the osseous portion of the petrous part of the temporal bone, positioned between the tympanic chamber and the floor of the inner ear canal. Irregular in shape, it is referred to as the labyrinth and comprises both the bony labyrinth and the membranous labyrinth [1]. The cochlea and vestibule within the bony labyrinth are essential for auditory and vestibular functions.

Endolymphatic hydrops (EH), also called membranous labyrinth hydrops, is a pathological condition marked by excessive endolymph accumulation within the membranous labyrinth. This abnormality results from either excessive endolymph production or impaired absorption, causing dilation of the endolymphatic space [2]. Elevated endolymph volume within the middle ear leads to endolymphatic effusion, followed by curvature of the vestibular and basilar membranes. Consequently, the stereocilia deviate from their normal position, reducing their sensitivity [3, 4]. Prolonged endolymphatic effusion may result in permanent hearing impairment, including stereocilia or synaptic loss, ultimately causing irreversible hearing loss [4]. 

EH is linked to episodic vertigo, fluctuating hearing loss, and various other conditions, such as Mondini malformation, Meniere’s disease, acute low-tone sensorineural hearing loss, and sudden sensorineural hearing loss [4–7]. Although EH holds clinical significance, its precise pathogenesis remains poorly understood.

Current therapeutic strategies for EH primarily involve pharmacological and surgical treatments, which are categorized based on their mechanisms of action [8]. The aim of the first strategy is to reduce the direct damage EH causes to the inner ear by employing treatments such as diuretics or endolymphatic sac decompression [9, 10]. The second strategy focuses on inhibiting abnormal peripheral signal transmission to the vestibular center, utilizing vestibular nerve inhibitors, chemical labyrinthectomy, or vestibular neurotomy [10, 11]. However, these treatments often demonstrate variable efficacy and carry potential adverse effects. Notably, although surgical interventions provide better vertigo control, they may also result in complications such as facial paralysis and hearing loss, negatively impacting patients’ quality of life [12]. As a result, pharmacological management remains the primary approach in clinical practice.

In traditional Chinese medicine (TCM), diseases induced by EH are classified as “ear dizziness.” The classic TCM text Danxi’s Mastery of Medicine states, “No phlegm, no dizziness.” Consequently, TCM treatments for ear dizziness have traditionally focused on resolving phlegm. Wendan decoction, a well-known formula for resolving phlegm, has been passed down through generations of physicians [13]. This formula comprises six herbs: *Pinelliae Rhizoma* [Araceae], *Poria* [Polyporaceae], *Citri Reticulatae Pericarpium* [Rutaceae], *Bambusae Caulis in Taenias* [Poaceae; bamboo shavings], *Citri Reticulatae Pericarpium* [Rutaceae], and *Glycyrrhizae Radix et Rhizoma* [Fabaceae] [14]. According to modern pharmacological research, Wendan decoction and its modified formulations prove to be effective in treating various conditions, including neurological, digestive, cardiovascular, and endocrine disorders [14–17]. Although Wendan decoction has been used clinically to manage Meniere’s syndrome, its therapeutic potential for EH remains underexplored due to limited experimental evidence.

The aim of this study is to address the existing gap by first characterizing the chemical composition of Wendan decoction and then evaluating its therapeutic effects on EH using an animal model. Furthermore, network pharmacology is employed to elucidate the potential mechanisms underlying the action of Wendan decoction. By identifying the pharmacological targets of Wendan decoction and integrating them with disease-associated targets of Meniere’s disease, the key signaling pathways involved in its therapeutic effects are uncovered in this study. This integrated approach offers a comprehensive framework for understanding the mechanism of action of Wendan decoction in treating EH and provides new insights into its clinical application for related disorders.

## Materials and methods

### Drug and disease targets identification

The Bioinformatics Analysis Tool for Molecular Mechanism of TCM (BATMAN-TCM; http://bionet.ncpsb.org.cn/batman-tcm/) was utilized to search for TCM components using the keywords “*Pinelliae Rhizoma*,” “*Poria*,” “*Citri Reticulatae Pericarpium*,” “*Bambusae Caulis in Taenias*,” “*Aurantii Fructus Immaturus*,” and “*Glycyrrhizae Radix et Rhizoma*.” Screening parameters included a score cutoff of ≥ 20 and a significance threshold of *p* < 0.05. Potential target genes associated with Meniere’s disease were retrieved from GeneCards (https://www.genecards.org), DisGeNet (https://www.disgenet.org/), and Online Mendelian Inheritance in Man (OMIM) (https://omim.org/) using the keyword “Meniere’s.”

### Gene function and pathway enrichment analysis

Gene Ontology (GO) analysis, encompassing biological processes, cellular components, and molecular functions, along with Kyoto Encyclopedia of Genes and Genomes (KEGG) pathway enrichment analysis, was conducted using Metascape (http://metascape.org) and the bioinformatics.com.cn platform. Statistical significance was set at *p* < 0.05.

### Animal ethics statement

Male guinea pigs (300 ± 10 g, *n* = 30) were obtained from Hangzhou Medical College and housed in an SPF-grade animal facility. All experimental protocols followed institutional guidelines for animal welfare and received approval from the Ethics Committee of Jiangyin Hospital of Traditional Chinese Medicine (No. 202231).

### Auditory brainstem response threshold detection

Guinea pigs were anesthetized with isoflurane (R510-22, RWD, China) and secured on the operating table. Subcutaneous electrodes were positioned bilaterally behind the ears (recording), at the frontal midline (reference), and dorsogluteally (ground). Auditory brainstem response (ABR) thresholds were measured using 19.1 Hz tone bursts, with a low-pass filter set at 1500 Hz and a high-pass filter at 150 Hz, incorporating 2000 signal averages. Stimulus intensity began at 80 dBnHL and decreased in 10 dB increments, followed by 5 dB steps near the threshold. The ABR threshold was defined as the lowest intensity that produced a reproducible Wave III response.

### Distortion product otoacoustic emission testing

Following ABR testing, the guinea pigs underwent distortion product otoacoustic emission (DPOAE) testing. A probe was secured in the external auditory canal, ensuring a sealed cavity during the procedure. Testing commenced once the guinea pigs were fully sedated (immobile) with stable spontaneous breathing. The primary frequencies used were f1 and f2 (f2/f1 = 1.22), with a measurement range of 2.0 to 10.0 kHz. The corresponding tone intensities were L1 (70 dBSPL) and L2 (65 dBSPL). Measurements were taken at six frequencies: 2222 Hz, 2963 Hz, 4444 Hz, 5714 Hz, 8000 Hz, and 10,000 Hz. The DPOAE amplitude was calculated as 2f1 - f2, and a signal-to-interference-plus-noise ratio (SINR) greater than 6 dBSPL was considered a passing result.

### Preparation of Wendan decoction granules

Wendan decoction granules (Tianjiang Pharmaceutical Co., Ltd., China) comprised *Pinelliae Rhizoma* (6 g), Poria (9 g), *Citri Reticulatae Pericarpium* (9 g), *Bambusae Caulis in Taenias* (9 g), *Aurantii Fructus Immaturus* (9 g), and *Glycyrrhizae Radix et Rhizoma* (3 g) [14]. The granules were dissolved in double-distilled water to prepare low- (6.1 g/kg), medium- (12.2 g/kg), and high-dose (24.4 g/kg) solutions.

### Ultra-high performance liquid chromatography and quadrupole time of flight mass spectrometry (UHPLC-QTOF/MS) analysis

Materials and Reagents: Chromatography-grade water and methanol were used in the experiment. Milli-Q filtered ultrapure water (Millipore, USA) was utilized. Mass spectrometry-grade acetonitrile was obtained from Fisher Scientific (Fair Lawn, New Jersey, USA), and mass spectrometry-grade formic acid was sourced from Sigma-Aldrich (St. Louis, Missouri, USA).

Sample Preparation: Wendan decoction (0.1 g) was ultrasonicated in 10 mL of 70% methanol for two 30-minute intervals. The resulting supernatants were combined, evaporated under nitrogen, reconstituted in water, and purified using a C18 solid-phase extraction column. Following centrifugation at 12,000 r/min for 10 min, 400 µL of supernatant was evaporated under nitrogen. Subsequently, 400 µL of 70% methanol was added. After another round of centrifugation at 13,000 r/min for 10 min, 2 µL of the resulting supernatant was aspirated and injected into a liquid chromatograph-mass spectrometer (LC-MS).

Chromatography: A Waters Acquity HSS T3 column (2.1 × 150 mm, 1.8 μm; 35 °C) was used. The mobile phases consisted of A: 0.1% formic acid in water and B: acetonitrile. The flow rate was set at 0.3 ml/min, and the injection volume was 2 µL. Elution gradients are presented in Table 1.

**Table 1:** Elution gradient

Time (min)	A%	B%
0	97	3
5	92	8
11	70	30
20	20	80
21	5	95
28	5	95
pre-equilibrium 6 min		

Mass spectrometry: Sample analysis was performed using the SCIEX Exion LC United X500B Q-TOF mass spectrometer (AB Sciex, Foster City, CA, USA). The positive and negative ion scanning modes of the electrospray ion source (ESI) are presented in Table 2, with the scanning range from m/z 100 to 1250.

**Table 2:** Mass spectrometry conditions

Ion mode	ESI (+)	ESI (-)
GS1	55	55
GS2	55	55
CUR	35	35
TEM	550	550
ISVF	5500	-4500
DP(MS)	60	-60
DP(MS/MS)	60	60
CE(MS/MS)	35	-35
CES	15	15

### Animal handling

Guinea pigs with baseline ABR thresholds < 40 dB SPL and passing DPOAE were randomly assigned to five groups (*n* = 6/group): Control (CON), Model, low-dose treatment (Model + L), medium-dose treatment (Model + M), and high-dose treatment (Model + H). EH was induced in all guinea pigs, except those in the CON group, through intraperitoneal injections of desmopressin acetate (6 µg/kg/d; 20211202, Hainan Zhonghe Pharmaceutical Co., Ltd, China) for 10 days. Starting from day 11, the treatment groups were administered oral Wendan decoction for 10 days.

### Behavioral test

At the conclusion of the treatment, guinea pigs from each group underwent the Rota-Rod test, which measures the duration an animal remains on a rotating circular wheel [18]. The aim of this experiment was to assess the animals’ motor coordination. The guinea pigs were initially placed on a rotarod fatigue tester (XR-6 C, Shanghai Xinruan Information Technology Co. Ltd, China), with the rotational speed set at 30 rpm for 2 min. After 2 days of training (3 trials/day), 3 tests were conducted on the third day. The final drop time (in seconds) was averaged across the three trials.

The guinea pigs were subsequently subjected to the swim test. The pool’s water level was set to 15 cm, and the water temperature was maintained at approximately 25 °C. After the guinea pigs were placed in the pool, their scores were recorded based on the following criteria: 0 points for swimming in the water, 1 point for swimming irregularly, 2 points for floating stationary, and 3 points for rolling underwater.

### Hematoxylin and eosin staining

Cochlear tissue was immersed in 4% paraformaldehyde and adequately dehydrated. Paraffin-embedded sections of the cochlea were prepared (4 μm). After deparaffinization, the sections were sequentially stained using hematoxylin and eosin solutions (Hematoxylin and Eosin (HE) Staining Kit, C0105M, Beyotime, China). The sections were observed under an OLYMPUS microscope (magnification: 40 ×; BY43, Japan), and the cross-sectional area of the middle and vestibular orders of each gyrus in the cochlear sections was determined using ImageJ image processing software (R value = area of the middle order / area of the middle order + area of the vestibular order).

### Organ of corti imaging

The cochlea was immersed in 2% glutaraldehyde (G6257, Sigma-Aldrich, USA) for fixation. After decalcification and fixation, the cochlea’s ossicles and vestibular membrane were removed to expose the inner or outer hair cells. Dehydrated and dried tissues were examined under a ZEISS scanning electron microscope (Sigma 300, Germany) to observe the cochlear Corti organ area. The numbers of inner and outer hair cells in a 140 μm basal segment of the basilar membrane were calculated [19]. Only hair cells with complete three-dimensional ciliary bundles and cuticular plates were counted.

### ELISA

The cochleae of guinea pigs were homogenized for ELISA analysis using the following kits: Cavia Interleukin 1 Beta (IL-1β) ELISA Kit (JL21516, China), Cavia Interleukin 6 (IL-6) ELISA Kit (Shanghai HengYuan Biotechnology Co., LTD, China), and Guinea pig TNF-α ELISA Kit (GOY-2922E, bio-goy, China). After the reaction was terminated, absorbance was measured using SpectraMax iD5 Microplate Readers (Molecular Devices, USA) at 450 nm.

### Quantitative real-time polymerase chain reaction

TRIzol reagent (15596026, Invitrogen, USA) was added to the cochlear homogenate to efficiently isolate RNA. RNA was reverse-transcribed to cDNA using the RevertAid cDNA Synthesis Kit (K1622, ThermoFisher, USA). The cDNA was mixed with pre-prepared gene primers (AQP2, P38MAPK, and GAPDH; Table 3) and SYBR Green (SR1110, Solarbio, China), and analyzed on a Fast7500 PCR instrument (ABI, USA). Relative mRNA levels were calculated based on the cycle threshold (CT) value measured by the instrument using the 2^−ΔΔCT^ method.

**Table 3:** Primers for qRT-PCR

Genes	Forward primer(5’-3’)	Reverse primer(5’-3’)
AQP2	GATCGCCGTGGCCTTTGGTCT	AGGGAGCGGGCTGGATTCAT
P38MAPK	CAGCTTCAGCAGATTATGCGT	AGCCACTGGTTCATCGTCAG
GAPDH	TGTGGGCATCAATGGATTTGG	ACACCATGTATTCCGGGTCAAT

### Immunohistochemical staining

The deparaffinized cochlea sections underwent antigen retrieval and permeabilization. To enhance detection and observation, the periphery of the cochlea was outlined using Liquid Blocker Super PAP Pen (P0139, Beyotime, China). Anti-AQP2 antibody (1:1000; ab199975, Abcam, USA) was applied dropwise within the outlined area to cover the entire cochlear tissue. The following day, diluted Goat Anti-Rabbit IgG H&L (HRP) (1:1000; ab6721, Abcam, USA) was incubated with the cochlea for 1 h. DAB working solution (P0202, Beyotime, China) was used for color development of positive expression. AQP2 positive expression (brown color) was observed using a microscope (magnification: 200×). Staining intensity: evaluation of the depth of staining in positive cells (representing the relative amount of AQP2). Typically classified into 4 grades: 0: No staining (negative); 1: Weakly positive (light brown); 2: Moderately positive (brown); 3: Strongly positive (dark brown).

### Statistical analyses

Statistical analysis of the data was performed using GraphPad Prism 8.0 software. Data were presented as mean ± standard deviation (SD). One-way ANOVA followed by Tukey’s post hoc multiple comparison tests was used to compare differences between groups. Results were considered statistically significant at *p* < 0.05.

## Results

### Characterization of the chemical composition of Wendan decoction

**Fig. 1:**
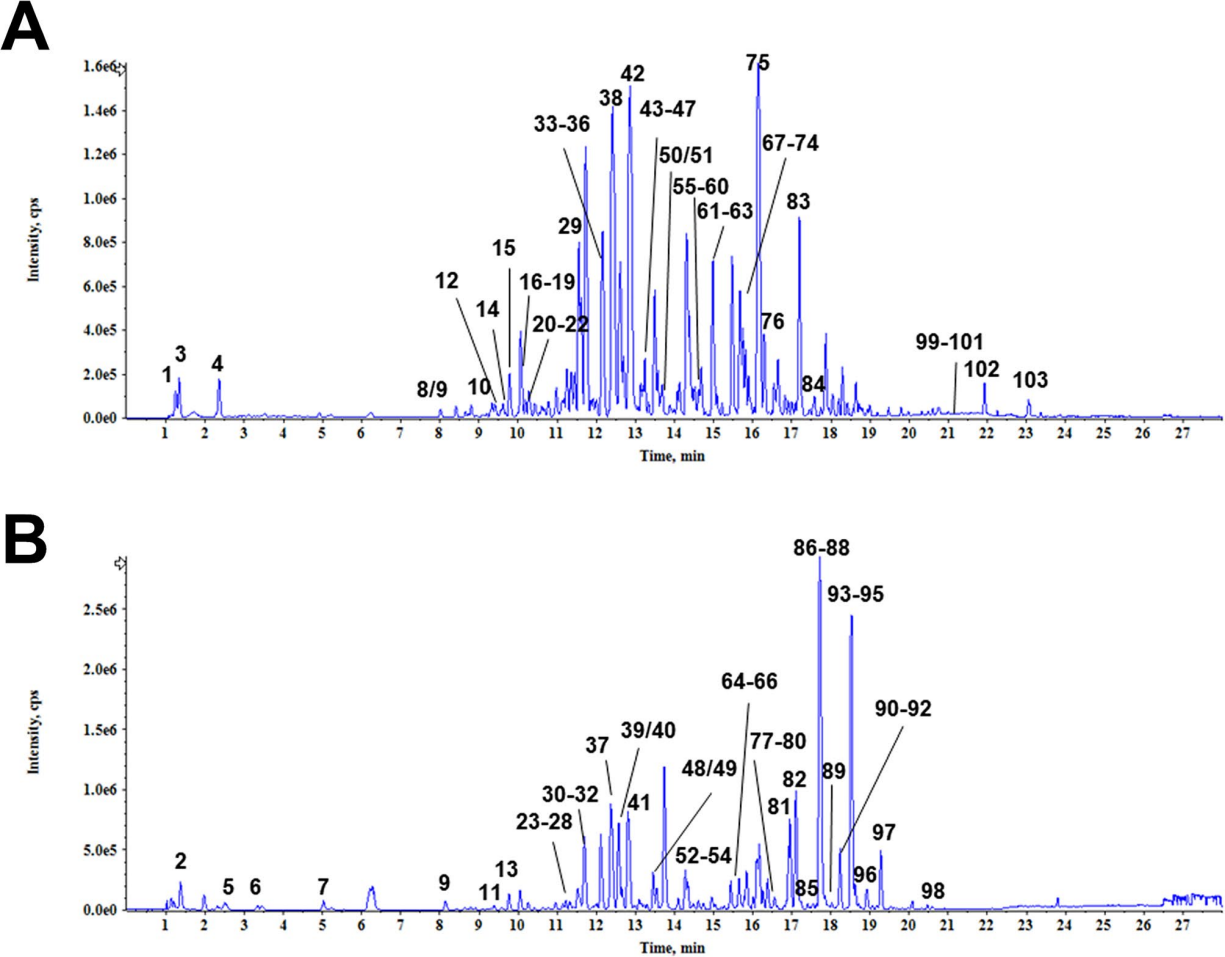
Analysis of the components of Wendan decoction. **(a)** BPC diagram in Negative ions mode **(b)** BPC diagram in Positive ions mode. BPC: Base Peak Chromatogram

**Fig. 2:**
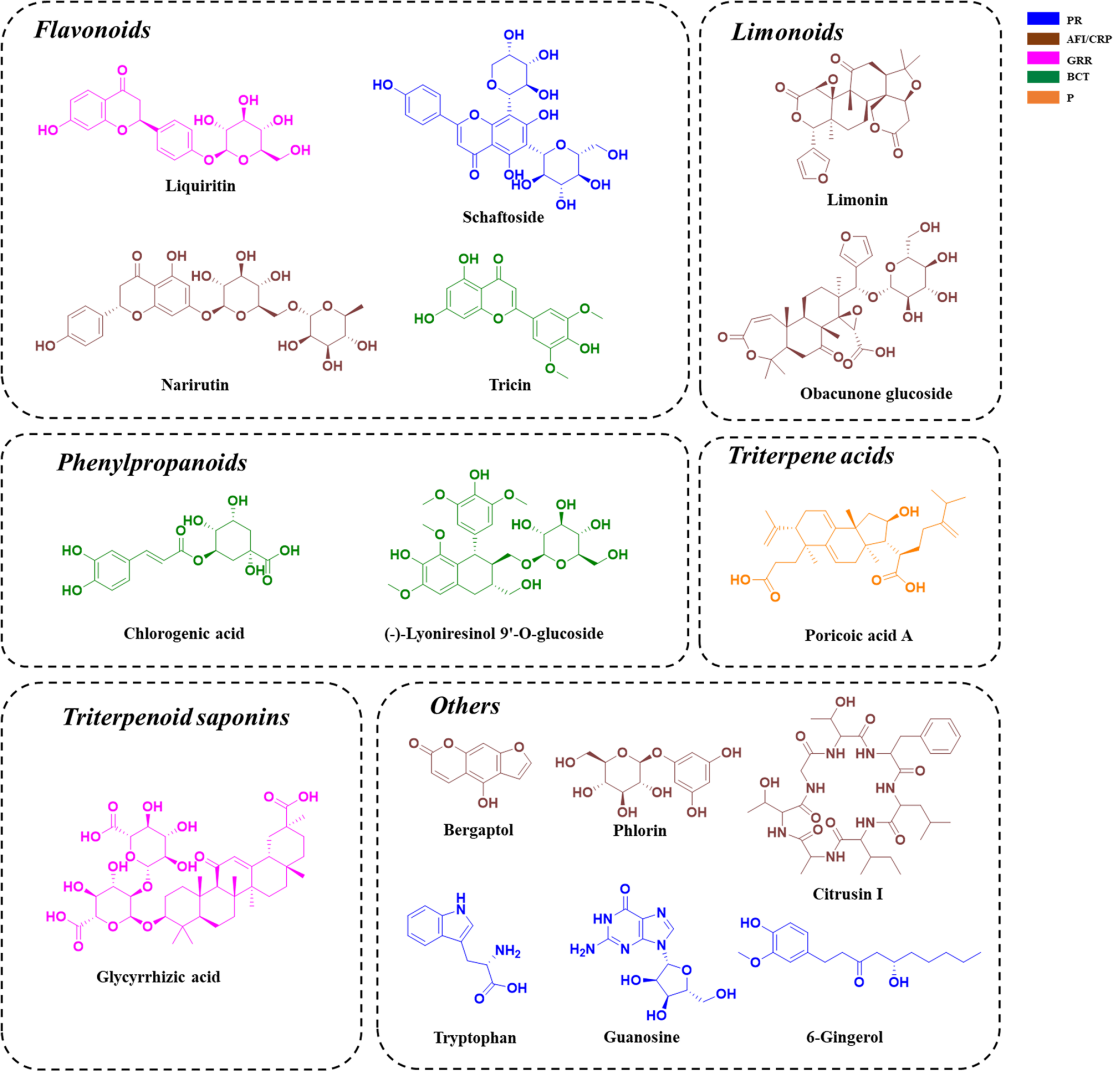
Structural formulae for representative chemical types. Different colors represent different sources: Blue: PR (Pinelliae Rhizoma); Brown: AFI (Aurantii Fructus Immaturus), CRP (Citri Reticulatae Pericarpium); Pink: GRR (Glycyrrhizae Radix et Rhizoma); Green: BCT (Bambusae Caulis in Taenias); Orange: P (Poria)

Wendan decoction is composed of six herbal components: *Pinelliae Rhizoma*,* Aurantii Fructus Immaturus*,* Citri Reticulatae Pericarpium*,* Glycyrrhizae Radix et Rhizoma*,* Bambusae Caulis in Taenias*, and *Poria*. Using the UHPLC-QTOF/MS strategy for the structural resolution and identification of complex components in TCM, the unknown components were categorized based on cleavage patterns and diagnostic ions characteristic of different structural types. A total of 103 components were identified in Wendan decoction, primarily consisting of flavonoids, triterpenoid saponins, phenylpropanoids, triterpenoic acids, and coumarins. *Pinelliae Rhizoma* mainly contained amino acids, nucleosides, and other common primary metabolites of plants. Flavonoids, such as Schaftoside, and phenylpropanoids, such as Ferulic acid, have been reported in *Pinelliae Rhizoma*. Compounds like 6-Gingerol were also detected but with lower response intensities. *Aurantii Fructus Immaturus* and *Citri Reticulatae Pericarpium*, originating from the same plant family, shared many overlapping compounds, predominantly flavonoids. Thirty-four compounds were identified in *Aurantii Fructus Immaturus*, and 36 in *Citri Reticulatae Pericarpium*. *Glycyrrhizae Radix et Rhizoma* contributed 34 compounds, mainly flavonoids and triterpenoid saponins. *Bambusae Caulis in Taenias* yielded 11 compounds, primarily phenylpropanoids, with 9’-O-glucoside of Lyoniresinol as a distinctive component. *Poria* contained six triterpenic acid compounds. The Base Peak Chromatogram (BPC) of the aqueous extract of Wendan decoction is presented in Fig. 1, with compound numbers labeled on the graph, and the corresponding chemical constituents are listed in Supplementary Table 1. The structural formulae of the representative chemical types are presented in Fig. 2.

### GO and pathway analysis of Wendan decoction

**Fig. 3:**
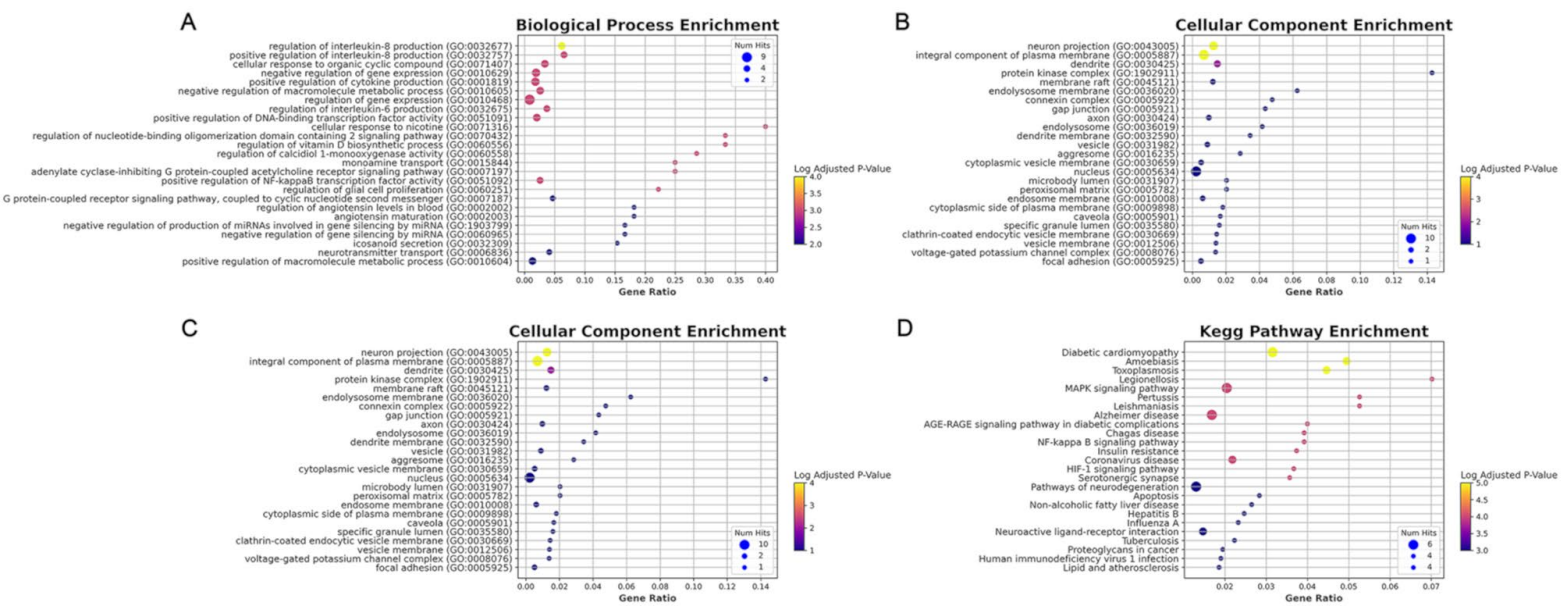
GO and KEGG pathway enrichment analysis. **(A)** Biological process categories, **(B)** Cellular component categories, **(C)** Molecular function categories, **(D)** KEGG pathway analysis were conducted using Metascape (http://metascape.org) and the bioinformatics.com.cn platform. Statistical significance was set at *p* < 0.05 GO: Gene Ontology; KEGG: Kyoto Encyclopedia of Genes and Genomes

Enrichment analysis of targets was conducted using the bioinformatics.com.cn platform. Significant enrichments (*p* < 0.05) were observed in biological processes (BP), cellular components (CC), molecular functions (MF), and KEGG pathways (Fig. 3). BP targets were primarily enriched in processes such as cellular response to lipids, response to lipopolysaccharide, regulation of secretion, response to hormones, and multicellular organism processes, among others as indicated in the results. CC targets were predominantly enriched in the basal part of the cell, receptor complexes, membrane rafts, and transport vesicles. MF targets were mainly enriched in receptor-ligand activity, nuclear receptor activity, hormone activity, heme binding, and G protein-coupled receptor binding. Based on the enriched biological process ontologies, the anti-Meniere’s effect of Wendan decoction is likely due to a complex synergistic effect of multiple biological processes. Pathway analysis revealed that 34 targets were involved in 10 KEGG pathways with significant *p* values (*p* < 0.05). As presented in the bubble diagram (Fig. 3D), the larger the bubble, the more genes that are enriched in the pathway, and the redder the color, the smaller the *p* value.

### Wendan decoction granules alleviates vestibular damage in EH model

**Fig. 4:**
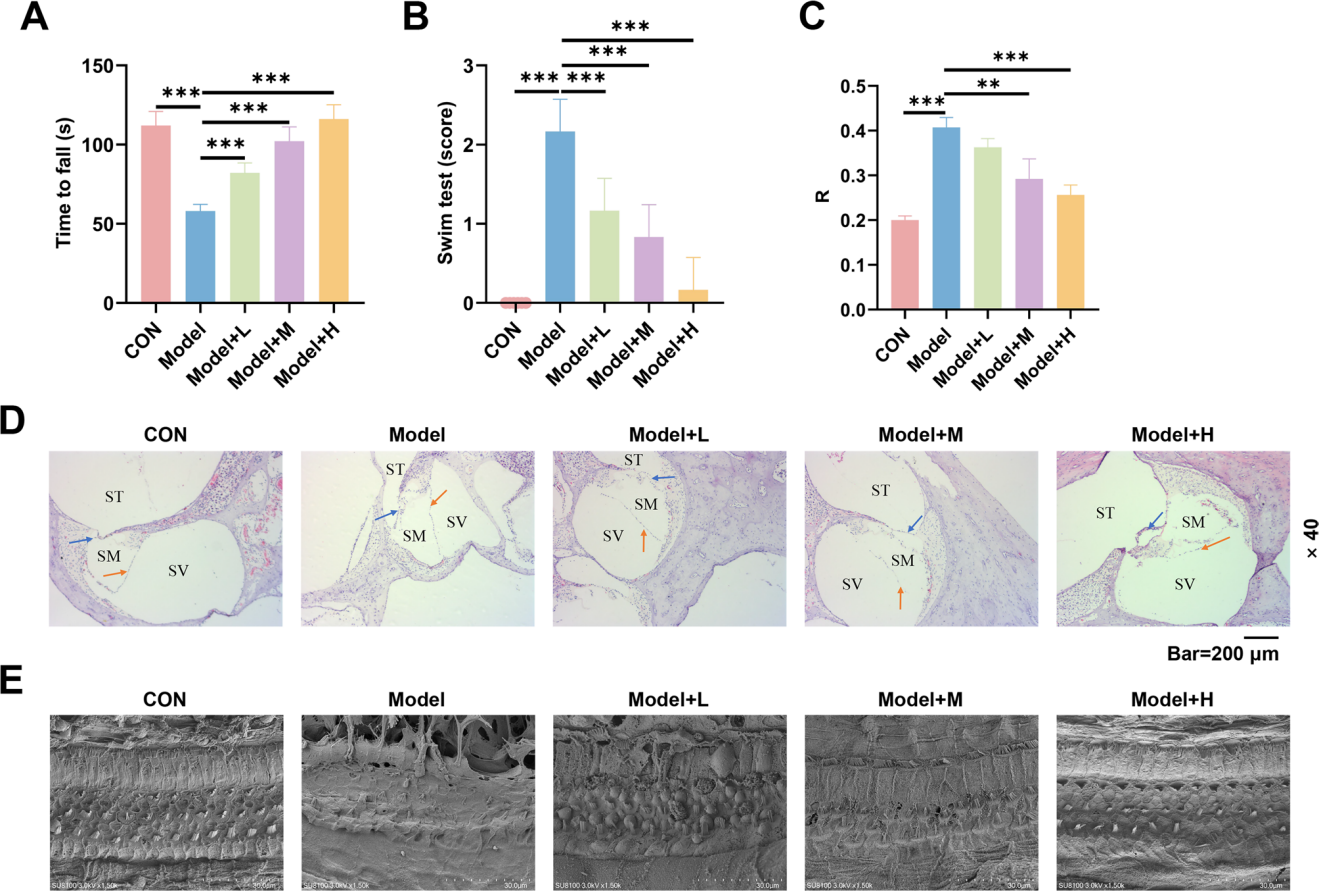
Wendan decoction granules mitigate vestibular damage induced by EH. Guinea pigs were randomly assigned to five groups (*n* = 6/group): Control (CON), Model, low-dose treatment (Model + L (6.1 g/kg Wendan decoction)), medium-dose treatment (Model + M (12.2 g/kg Wendan decoction)), and high-dose treatment (Model + H (24.4 g/kg Wendan decoction)). **(A-B)** The Rota-Rod and swim tests were used to assess the effects of Wendan decoction granules on the motor coordination of guinea pigs. **(C-D)** Fluid accumulation in the membranous labyrinth of guinea pigs was evaluated through hematoxylin and eosin (HE) staining. Bar = 200 μm, Magnification: 40×. Blue arrow: Basement membrane; Orange arrow: Reissner’s membrane; SM: Scala media; SV: Scala vestibuli; ST: Scala tympani. **(E)** Scanning electron microscopy was employed to observe the effect of Wendan decoction granules on the structure of the cochlear Corti organ. All experiments were repeated three times to average. Significant differences between groups are indicated by horizontal lines with the level of significance indicated by asterisk(s). ^***^p < 0.001

Behavioral experiments revealed the therapeutic efficacy of Wendan decoction granules. In the Rota-Rod test, guinea pigs in the model group remained on the rotating rod for a shorter duration (*p* < 0.001, Fig. 4A) and had a higher tendency to fall off. In the swim test, guinea pigs in the CON group scored 0, while those in the model group scored higher (*p* < 0.001, Fig. 4B). Treatment with Wendan decoction granules progressively increased Rota-Rod latencies and reduced swim test scores (*p* < 0.001, Fig. 4A-B), indicating an improvement in vestibular function. The degree of fluid accumulation in the membranous labyrinth of guinea pigs was assessed further. Histological analysis using HE staining demonstrated reduced fluid accumulation in the membranous labyrinth of treated groups compared to the model group (*p* < 0.01, Fig. 4C-D). Compared to the CON group, the model group exhibited severe loss of outer hair cells in the cochlear Corti organ, with minimal changes in inner hair cells (Fig. 4E). After treatment, particularly in the high-dose group, the loss of outer hair cells in the cochlear Corti organ region of guinea pigs was reduced (Fig. 4D).

### Wendan decoction reduces inflammatory factor release and AQP2 expression

**Fig. 5:**
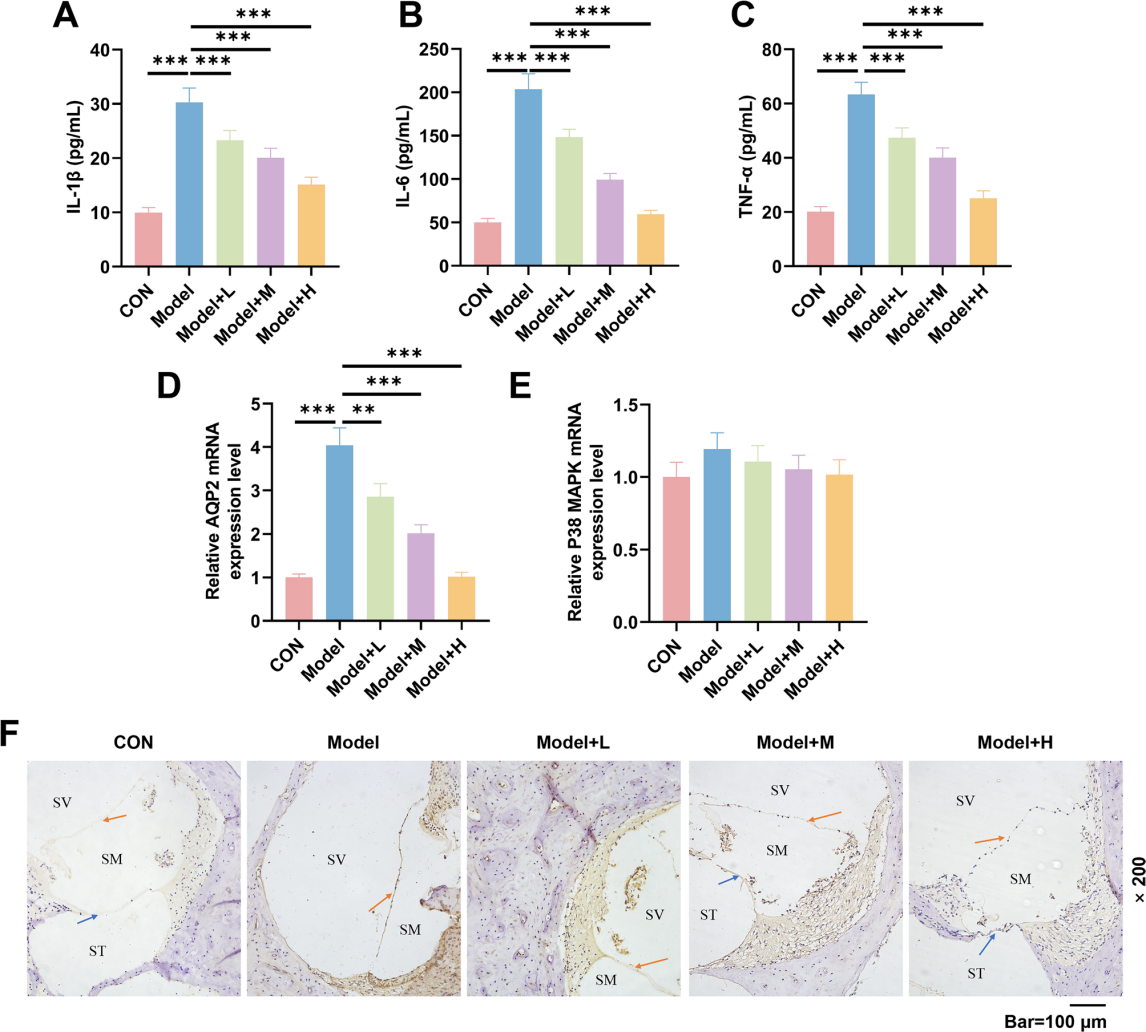
Wendan decoction granules decreased the release of inflammatory factors and AQP2 protein expression in the EH model. Guinea pigs were randomly assigned to five groups (*n* = 6/group): Control (CON), Model, low-dose treatment (Model + L (6.1 g/kg Wendan decoction)), medium-dose treatment (Model + M (12.2 g/kg Wendan decoction)), and high-dose treatment (Model + H (24.4 g/kg Wendan decoction)). **(A-C)** The levels of pro-inflammatory factors in the cochlea were measured using ELISA to assess the effects of Wendan decoction granules. **(D-E)** The expressions of AQP2 and P38MAPK in the cochlea were analyzed using qRT-PCR. GAPDH served as the internal reference gene. **(F)** The impact of Wendan decoction granules on AQP2 protein expression in the cochlea was evaluated by immunohistochemical staining. Bar = 100 μm, Magnification: 200 ×. Blue arrow: Basement membrane; Orange arrow: Reissner’s membrane; SM: Scala media; SV: Scala vestibuli; ST: Scala tympani. All experiments were repeated three times to average. Significant differences between groups are indicated by horizontal lines with the level of significance indicated by asterisk(s). ^**^p < 0.01, ^***^p < 0.001

Serum levels of proinflammatory factors IL-1β, IL-6, and TNF-α were significantly elevated (*p* < 0.001, Fig. 5A-C) in the model group, indicating that EH induced a strong inflammatory response in the cochlea. However, following treatment with Wendan decoction granules, the levels of IL-1β, IL-6, and TNF-α were significantly reduced (*p* < 0.001, Fig. 5A-C). Changes in AQP2 and P38MAPK signaling were further analyzed by qRT-PCR experiments. The results revealed that AQP2 was highly expressed in the cochlea of model guinea pigs (*p* < 0.001, Fig. 5D), while P38MAPK expression demonstrated a tendency to elevate, but the difference was not statistically significant (Fig. 5E). In the treatment group, the elevated AQP2 signals in the cochlea of model guinea pigs were downregulated (*p* < 0.01, Fig. 5D), along with a weakened P38MAPK signal (Fig. 5E). Immunohistochemical staining results (Fig. 5F) demonstrated that the area of brown positive expression was larger in the model group compared to the CON group, indicating upregulated AQP2 protein expression. However, in the treatment group, the expression of AQP2 protein decreased, indicating that Wendan decoction inhibits abnormal AQP2 activation in the cochlea.

## Discussion

The main pathological feature of Meniere’s disease is EH, which leads to recurrent vertigo, tinnitus and fluctuating hearing loss [20]. Hearing loss is one of the core symptoms, usually starting at low frequencies and gradually affecting high frequencies, which can eventually lead to permanent deafness. The severity of EH is associated with hearing loss [21]. Although the etiology of EH may be related to autoimmunity, allergies, genetics, and infections, the pathogenesis and therapeutic interventions for EH remain incompletely understood. Therefore, the development of a stable and physiologically relevant animal model is crucial for investigating the pathogenesis and therapeutic interventions of EH. The cochlea of guinea pigs is surrounded by less soft tissue, and the cochlear-to-body weight ratio is relatively large. Additionally, since the guinea pig’s cochlear structure closely resembles that of humans, as demonstrated by MRI, guinea pigs were chosen for this study due to their well-established utility in auditory research [22]. Various methods have been demonstrated to induce EH, including surgical obstruction of the endolymphatic sac, immunoassay, desmopressin injection after electrocautery of the endolymphatic sac, and systemic vasopressin administration [23–25]. To induce EH quickly and effectively while minimizing guinea pig suffering, intraperitoneal injection of desmopressin acetate was selected in this study. Desmopressin acetate is a vasopressin derivative with diuretic and vasopressor effects [26]. Vasopressin, a natriuretic peptide hormone secreted by neuronal cells in the supraoptic and paraventricular nuclei of the hypothalamus, primarily increases the permeability of the distal convoluted tubule and collecting ducts to water, facilitating water uptake [27]. Kitano et al. demonstrated that vasopressin is closely associated with endolymphatic fluid accumulation and that activation of vasopressin-AQP2 leads to excessive endolymphatic fluid production [28, 29]. Takeda et al. successfully induced an EH model in guinea pigs by subcutaneously implanting a vasopressin micropump one week later [30]. The findings support these mechanisms, as desmopressin-treated guinea pigs displayed significant vestibular dysfunction and cochlear fluid retention, as confirmed by behavioral tests and histopathological analysis. These results validate the efficacy of the model in replicating key features of EH while minimizing procedural invasiveness.

Behavioral assessments, including the Rota-Rod and swim tests, provided valuable insights into vestibular impairment. The observed reduction in motor coordination and balance in model animals corresponds with the pathophysiological distortion of the basilar membranes caused by endolymphatic expansion [3, 4]. Treatment with Wendan decoction alleviated these deficits, highlighting its potential to restore vestibular function. The improvement is particularly significant due to the vestibule’s central role in maintaining equilibrium [31]. This also facilitated the intuitive assessment of the therapeutic effects of Wendan decoction on the pathological behavior of guinea pigs.

In addition to behavioral and pathological changes, inflammatory indicators in the cochlea of guinea pigs were compared before and after treatment. The immunoinflammatory mechanism is one of the primary pathogenic mechanisms of endolymphatic effusion [18, 32]. Elevated levels of IL-1β, IL-1RA, TNF-α, and IL-6 in model animals reflect the inflammatory environment associated with Meniere’s pathology [33]. Wendan decoction effectively reduced the levels of pro-inflammatory factors IL-1β, IL-6, and TNF-α, with similar anti-inflammatory effects reported in depression treatment, underscoring its multimodal therapeutic action [34, 35]. Furthermore, higher activity of AQP2 protein was observed in the cochlea of the model group.

Aquaporins (AQPs) are crucial proteins identified in recent decades as regulators of aqueous homeostasis in cells involved in inflammatory responses [36]. Eight AQP isoforms have been identified in the membranous labyrinth of the inner ear, and AQPs play an essential physiological role in the volume regulation of the outer and inner lymph [37]. AQP2, a water channel protein, is expressed in fluid-transporting cells, such as endolymphatic sac epithelial cells, in addition to the kidney, and plays a significant role in fluid transport in the inner ear [38]. Vasopressin-induced EH primarily occurs through the regulation of the cAMP-AQP2 signaling pathway [37]. Pathological elevation of AQP2 signaling due to vasopressin can lead to increased opening of aqueous channels, thereby promoting the development of EH [39]. In this study, Wendan decoction blocked the aberrant activation of AQP2, indicating that Wendan decoction alleviates vasopressin-mediated EH by modulating cochlear water channel proteins. This is possibly the first report linking Wendan decoction to AQP2 modulation, broadening its pharmacological profile beyond traditional applications.

P38MAPK is a member of the highly conserved MAPK family and has been implicated in AQP2 regulation through proteasomal degradation in renal models [40]. The desmopressin acetate used in the EH guinea pig model in this study is a direct analogue of AVP. No significant changes in P38MAPK mRNA levels are observed in this study. Future studies should investigate the interaction between P38MAPK and AQP2 in cochlear tissues to clarify the role of this pathway in EH.

In conclusion, the chemical composition of Wendan decoction is outlined in this study, which demonstrates its effectiveness in alleviating EH through anti-inflammatory and AQP2-inhibitory mechanisms. These findings highlight the therapeutic potential of TCM in addressing complex auditory-vestibular disorders. Given TCM’s favorable safety profile and holistic action, further investigation into Wendan decoction and its integration with conventional therapies may offer innovative approaches to improving patient outcomes. Future research should focus on translational studies to validate these preclinical findings and optimize dosage protocols for clinical use.

## Supplementary Information

Below is the link to the electronic supplementary material.

Supplementary Material 1

## Data Availability

No datasets were generated or analysed during the current study.
