# Supplementary material for: Effect of Wendan decoction granules on preventing endolymphatic hydrops and protecting vestibular function in Guinea pigs
Source: Hereditas. 2025 Jul 26;162:145. doi: 10.1186/s41065-025-00516-2 (PMC12296600; doi:10.1186/s41065-025-00516-2)
Supplement: Supplementary file 1 — Supplementary Material 1 [file 41065_2025_516_MOESM1_ESM.docx]

Supplementary Table 1. Chemical composition characterisation results

| **NO.** | **Compound** | **Formula** | **RT (min)** | **Ion mode** | **Precursor Mass** | **Found At Mass** | **Mass Error (ppm)** | **Fragments** | **Class** | **Source** |
| --- | --- | --- | --- | --- | --- | --- | --- | --- | --- | --- |
| 1 | L-Arginine* | C_6_H_14_N_4_O_2_ | 1.13 | [M+H]^+^ | 175.1190 | 175.1191 | 0.9 | 130.0965, 116.0699, 70.0651, 60.0556, 43.0292 | Amino acids | ALL |
|  |  |  |  | [M-H]^-^ | 173.1044 | 173.1046 | 1.2 | 131.0824, 41.0151 |  |  |
| 2 | Sucrose | C_12_H_22_O_11_ | 1.33 | [M+NH_4_]^+^ | 360.1500 | 360.1500 | 0.0 | 163.0602, 145.0491, 127.0386, 115.0386, 109.0273, 99.0441, 97.0288, 85.0281, | Glycosides | ALL |
|  |  |  |  | [M+FA-H]^-^ | 387.1144 | 387.1146 | 0.4 | 341.1085, 179.0552, 161.0454, 149.0451, 143.0355, 119.0343, 113.0240, 101.0241, |  |  |
| 3 | Quinic acid* | C_7_H_12_O_6_ | 1.35 | [M-H]^-^ | 191.0561 | 191.0564 | 1.4 | 111.0448, 109.0319, 87.0088, 85.0304, 41.0039 | Organic acids | AFI |
| 4 | Phlorin | C_12_H_16_O_8_ | 2.36 | [M+H]^+^ | 289.0918 | 289.0918 | 0.0 | 165.0544, 127.0398, 109.0273, 85.0291, 81.0345 | Glycosides | AFI |
|  |  |  |  | [M+FA-H]^-^ | 333.0827 | 333.0830 | 0.7 | 149.0239, 125.0244, 83.0144, 81.0354, 57.0350, 44.9992 |  |  |
| 5 | Adenosine* | C_10_H_13_N_5_O_4_ | 2.51 | [M+H]^+^ | 268.1040 | 268.1039 | -0.5 | 136.0618, 119.0351, 85.0272, 55.0170, 43.0177 | Nucleosides | PR |
| 6 | Guanosine* | C_10_H_13_N_5_O_5_ | 3.35 | [M+H]^+^ | 284.0989 | 284.0989 | -0.1 | 152.0572, 135.0297, 110.0354, 88.0759, 82.0406 | Nucleosides | PR |
| 7 | L-Phenylalanine* | C_9_H_11_NO_2_ | 4.92 | [M+H]^+^ | 166.0863 | 166.0862 | -0.4 | 120.0806, 103.0541, 102.0457, 93.0702, 91.0537, 79.0548, 77.0385 | Amino acids | ALL |
|  |  |  |  | [M-H]^-^ | 164.0717 | 164.0719 | 1.5 | 147.0441, 103.0550, 91.0560, 72.0092 |  |  |
| 8 | Neochlorogenic acid | C_16_H_18_O_9_ | 7.90 | [M-H]^-^ | 353.0878 | 353.0884 | 1.7 | 191.0549, 179.0362, 135.0444, 134.0351 | Phenylpropanoids | BCT |
| 9 | Tryptophan* | C_11_H_12_N_2_O_2_ | 8.02 | [M-H]^-^ | 203.0826 | 203.0827 | 0.6 | 142.0659, 116.0503, 74.0246 | Amino acids | PR |
| 10 | Chlorogenic acid | C_16_H_18_O_9_ | 9.29 | [M-H]^-^ | 353.0878 | 353.0882 | 1.1 | 191.0561, 173.0428, 161.0246, 127.0389, 85.0294 | Phenylpropanoids | BCT |
| 11 | Fabiatrin isomer | C_21_H_26_O_13_ | 9.41 | [M+H]^+^ | 487.1446 | 487.1443 | -0.6 | 341.0859, 179.0336, 129.0533, 85.0292, 71.0496 | Glycosides | AFI |
|  |  |  |  | [M+FA-H]^-^ | 531.1355 | 531.1355 | -0.1 | 191.0375, 177.0186, 135.0085, 133.0292, 89.0396 |  |  |
| 12 | Cryptochlorogenic acid | C_16_H_18_O_9_ | 9.49 | [M-H]^-^ | 353.0878 | 353.0881 | 0.8 | 191.0559, 179.0358, 173.0453, 161.0218, 135.0441 | Phenylpropanoids | BCT |
| 13 | Lucenin-2 | C_27_H_30_O_16_ | 9.59 | [M+H]^+^ | 611.1607 | 611.1606 | 0.0 | 611.1580, 593.1525, 575.1373, 557.1277, 497.1085, 491.1167, 473.1107, 455.0970, | Flavonoids | AFI/CRP |
|  |  |  |  | [M-H]^-^ | 609.1461 | 609.1462 | 0.2 | 609.1442, 519.1135, 489.1029, 429.0807, 411.0712, 399.0708, 369.0603 |  |  |
| 14 | Liquiritigenin 4', 7-diglucoside | C_27_H_32_O_14_ | 9.62 | [M+FA-H]^-^ | 625.1774 | 625.1774 | -0.1 | 418.1257, 417.1188, 255.0650, 135.0098, 119.0509 | Flavonoids | GRR |
| 15 | Fabiatrin | C_21_H_26_O_13_ | 9.79 | [M+H]^+^ | 487.1446 | 487.1443 | -0.6 | 341.0880, 179.0337, 147.0654, 129.0557, 85.0283, 71.0497 | Glycosides | AFI |
|  |  |  |  | [M+FA-H]^-^ | 531.1355 | 531.1356 | 0.1 | 485.1306, 365.0881, 321.0604, 219.0298, 177.0191 |  |  |
| 16 | Caffeic Acid | C_9_H_8_O_4_ | 10.00 | [M-H]^-^ | 179.0350 | 179.0351 | 0.6 | 135.0442, 134.0384, 108.0223, 106.0413, 91.0560 | Phenylpropanoids | BCT |
| 17 | Vicenin-2 | C_27_H_30_O_15_ | 10.05 | [M+H]^+^ | 595.1657 | 595.1653 | -0.8 | 595.1652, 577.1559, 559.1458, 541.1340, 529.1327, 523.1230, 511.1213, 505.1118, | Flavonoids | AFI/CRP |
|  |  |  |  | [M-H]^-^ | 593.1512 | 593.1509 | -0.5 | 593.1500, 575.1419, 533.1274, 515.1193, 503.1202, 485.1069, 473.1075, 455.1005, |  |  |
| 18 | Diosmetin-6, 8-di-C-glucoside | C_28_H_32_O_16_ | 10.26 | [M+H]^+^ | 625.1763 | 625.1758 | -0.8 | 625.1741, 607.1603, 589.1548, 571.1447, 559.1461, 553.1286, 541.1320, 529.1307, | Flavonoids | CRP |
|  |  |  |  | [M-H]^-^ | 623.1618 | 623.1614 | -0.7 | 623.1606, 533.1276, 503.1181, 443.0983, 413.0846, 383.0764, 312.0628 |  |  |
| 19 | Narirutin 4’-glucoside | C_33_H_42_O_19_ | 10.28 | [M+H]^+^ | 743.2393 | 743.2391 | -0.2 | 581.1769, 579.1715, 527.1553, 383.1079, 273.0739 | Flavonoids | CRP |
|  |  |  |  | [M+FA-H]^-^ | 787.2302 | 787.2300 | -0.3 | 741.2261, 579.1730, 434.1183, 433.1135, 271.0609 |  |  |
| 20 | Schaftoside* | C_26_H_28_O_14_ | 10.66 | [M+H]^+^ | 565.1552 | 565.1550 | -0.4 | 565.1545, 547.1480, 529.1325, 511.1234, 481.1160, 427.1010, 409.0961, 391.0815, | Flavonoids | PR/GRR |
|  |  |  |  | [M-H]^-^ | 563.1406 | 563.1405 | -0.2 | 563.1415, 473.1074, 443.1002, 383.0762, 353.0657 |  |  |
| 21 | 3-O-Feruloylquinic acid | C_17_H_20_O_9_ | 10.78 | [M+H]^+^ | 369.1180 | 369.1179 | -0.4 | 177.0541, 149.0590, 145.0283, 117.0345, 89.0380 | Phenylpropanoids | BCT |
|  |  |  |  | [M-H]^-^ | 367.1035 | 367.1034 | -0.1 | 193.0508, 191.0560, 173.0455, 134.0361, 111.0458, 93.0350, 87.0087 |  |  |
| 22 | (-)-Lyoniresinol 9'-O-glucoside | C_28_H_38_O_13_ | 10.98 | [M+FA-H]^-^ | 627.2294 | 627.2294 | 0.0 | 581.2234, 566.1989, 419.1717, 405.1533, 404.1498, 401.1570, 386.1396, 373.1289, | Phenylpropanoids | BCT |
| 23 | Naringin 4'-glucoside | C_33_H_42_O_19_ | 11.15 | [M+H]^+^ | 743.2393 | 743.2388 | -0.7 | 435.1277, 419.1305, 417.1152, 339.0861, 315.0859, 309.1180, 274.0784, 273.0760, | Flavonoids | CRP |
|  |  |  |  | [M-H]^-^ | 741.2248 | 741.2248 | 0.1 | 742.2239, 741.2238, 621.1678, 271.0594, 151.0028 |  |  |
| 24 | Violanthin | C_27_H_30_O_14_ | 11.23 | [M+H]^+^ | 579.1708 | 579.1703 | -0.9 | 579.1647, 561.1593, 543.1458, 525.1337, 441.1197, 423.1040, 405.0942, 393.0955, | Flavonoids | GRR |
|  |  |  |  | [M-H]^-^ | 577.1563 | 577.1564 | 0.1 | 577.1552, 473.1100, 457.1139, 383.0785, 353.0679 |  |  |
| 25 | ethyl-p-coumarate | C_11_H_12_O_3_ | 11.34 | [M+H]^+^ | 193.0859 | 193.0857 | -1.0 | 133.0644, 118.0424, 115.0542, 105.0692, 103.0543, 90.0470, 89.0381, 79.0549, | Phenylpropanoids | BCT |
| 26 | Neoeriocitrin | C_27_H_32_O_15_ | 11.36 | [M+H]^+^ | 597.1814 | 597.1807 | -1.2 | 451.1215, 435.1281, 417.1218, 399.1107, 381.0966, 355.0822, 331.0800, 289.0713, | Flavonoids | AFI/CRP |
|  |  |  |  | [M-H]^-^ | 595.1668 | 595.1667 | -0.2 | 595.1645, 549.1647, 459.1140, 311.0545, 287.0547, 255.0667, 175.0022, 151.0030, |  |  |
| 27 | Rutin* | C_27_H_30_O_16_ | 11.42 | [M+H]^+^ | 611.1607 | 611.1601 | -0.9 | 303.0491, 147.0654, 129.0534, 85.0273, 71.0497 | Flavonoids | AFI/CRP |
|  |  |  |  | [M-H]^-^ | 609.1461 | 609.1465 | 0.6 | 609.1451, 343.0478, 301.0341, 300.0261, 178.9977 |  |  |
| 28 | p-Coumaric acid* | C_9_H_8_O_3_ | 11.45 | [M+H]^+^ | 165.0546 | 165.0546 | -0.3 | 147.0456, 119.0499, 91.0547, 77.0395, 65.0395 | Phenylpropanoids | BCT |
|  |  |  |  | [M-H]^-^ | 163.0401 | 163.0402 | 0.7 | 119.0496, 117.0346, 116.0270, 93.0349, 91.0559 |  |  |
| 29 | Liquiritin apioside* | C_26_H_30_O_13_ | 11.53 | [M+H]^+^ | 551.1759 | 551.1755 | -0.7 | 257.0815, 239.0688, 147.0432, 145.0507, 137.0243, 133.0491, 127.0401, 115.0389, | Flavonoids | GRR |
|  |  |  |  | [M-H]^-^ | 549.1614 | 549.1614 | 0.2 | 550.1591, 549.1594, 429.1169, 417.1206, 399.1078, 297.0751, 269.0795, 255.0648, |  |  |
| 30 | Eriocitrin | C_27_H_32_O_15_ | 11.68 | [M+H]^+^ | 597.1814 | 597.1816 | 0.3 | 451.1251, 435.1274, 433.1101, 417.1212, 399.1060, 381.0980, 355.0817, 331.0795, | Flavonoids | AFI/CRP |
|  |  |  |  | [M-H]^-^ | 595.1668 | 595.1669 | 0.2 | 595.1645, 577.1551, 459.1139, 441.0993, 433.1354, 357.0816, 339.0708, 329.0659, |  |  |
| 31 | Liquiritin* | C_21_H_22_O_9_ | 11.70 | [M+NH_4_]^+^ | 436.1602 | 436.1604 | 0.3 | 257.0797, 242.0563, 239.0703, 211.0760, 163.0384, 147.0444, 145.0507, 137.0230, | Flavonoids | GRR |
|  |  |  |  | [M-H]^-^ | 417.1191 | 417.1187 | -0.9 | 417.1166, 256.0679, 255.0649, 254.0574, 213.0557, 149.0238, 148.0168, 145.0297, |  |  |
| 32 | Syringaldehyde | C_9_H_10_O_4_ | 11.75 | [M+H]^+^ | 183.0652 | 183.0651 | -0.7 | 95.0503, 77.0386, 56.9422, 55.0171, 39.0219 | Phenols | BCT |
| 33 | Deacetyl nomilinic acid glucoside | C_32_H_46_O_15_ | 11.85 | [M-H]^-^ | 669.2764 | 669.2760 | -0.6 | 669.2721, 609.2560, 101.0251, 89.0252 | Limonoids | AFI |
| 34 | Scoparin | C_22_H_22_O_11_ | 11.92 | [M+H]^+^ | 463.1235 | 463.1231 | -0.7 | 463.1207, 445.1128, 427.0971, 409.0945, 397.0911, 367.0804, 343.0809, 325.0721, | Flavonoids | AFI |
|  |  |  |  | [M-H]^-^ | 461.1089 | 461.1087 | -0.5 | 461.1115, 371.0761, 341.0666, 313.0727, 298.0484, 297.0401 |  |  |
| 35 | Ferulic acid* | C_10_H_10_O_4_ | 12.00 | [M+H]^+^ | 195.0652 | 195.0651 | -0.5 | 177.0540, 149.0602, 145.0283, 134.0356, 117.0333, 106.0417, 91.0546, 89.0389, | Phenylpropanoids | PR/BCT |
|  |  |  |  | [M-H]^-^ | 193.0506 | 193.0508 | 0.7 | 178.0256, 134.0370, 133.0291, 105.0340, 89.0395 |  |  |
| 36 | Narirutin* | C_27_H_32_O_14_ | 12.12 | [M+H]^+^ | 581.1865 | 581.1863 | -0.4 | 435.1282, 419.1351, 417.1177, 401.1230, 399.1087, 383.1130, 365.1012, 339.0864, | Flavonoids | AFI/CRP |
|  |  |  |  | [M+FA-H]^-^ | 625.1774 | 625.1778 | 0.6 | 625.1825, 580.1790, 579.1720, 459.1122, 325.0720, 313.0732, 295.0624, 285.0777, |  |  |
| 37 | Apigenin 7-O-neohesperidoside | C_27_H_30_O_14_ | 12.33 | [M+H]^+^ | 579.1708 | 579.1704 | -0.8 | 579.1698, 273.0742, 271.0598, 270.1652, 147.0653 | Flavonoids | CRP |
|  |  |  |  | [M-H]^-^ | 577.1563 | 577.1565 | 0.3 | 577.1574, 431.1037, 269.0445, 268.0382, 149.0226 |  |  |
| 38 | Naringin* | C_27_H_32_O_14_ | 12.38 | [M+H]^+^ | 581.1865 | 581.1859 | -0.9 | 435.1271, 419.1341, 417.1188, 401.1220, 399.1057, 383.1120, 381.0957, 369.0960, | Flavonoids | AFI |
|  |  |  |  | [M-H]^-^ | 579.1719 | 579.1717 | -0.3 | 580.1716, 579.1720, 459.1144, 441.1040, 433.1358, 417.1235, 415.1050, 373.0937, |  |  |
| 39 | Hesperidin* | C_28_H_34_O_15_ | 12.57 | [M+H]^+^ | 611.1970 | 611.1964 | -1.1 | 465.1381, 449.1445, 447.1273, 431.1356, 429.1191, 413.1259, 411.1085, 395.1130, | Flavonoids | AFI/CRP |
|  |  |  |  | [M-H]^-^ | 609.1825 | 609.1819 | -0.9 | 609.1806, 343.0839, 325.0718, 301.0714, 286.0481, 283.0607, 258.0537, 257.0812, |  |  |
| 40 | Diosmin | C_28_H_32_O_15_ | 12.64 | [M+H]^+^ | 609.1814 | 609.1814 | -0.1 | 609.1800, 301.0700, 300.0653, 286.0465, 129.0557 | Flavonoids | CRP |
|  |  |  |  | [M-H]^-^ | 607.1668 | 607.1674 | 0.9 | 607.1682, 341.0633, 299.0552, 284.0331 |  |  |
| 41 | Choerospondin* | C_21_H_22_O_10_ | 12.73 | [M+H]^+^ | 435.1286 | 435.1284 | -0.4 | 273.0761, 153.0189, 147.0444, 123.0448, 119.0499 | Flavonoids | GRR |
|  |  |  |  | [M-H]^-^ | 433.1140 | 433.1141 | 0.1 | 271.0615, 177.0203, 151.0044, 119.0499, 107.0132, 93.0341 |  |  |
| 42 | Neohesperidin* | C_28_H_34_O_15_ | 12.82 | [M+H]^+^ | 611.1970 | 611.1967 | -0.6 | 465.1381, 449.1424, 447.1294, 431.1335, 429.1169, 413.1238, 411.1085, 399.1060, | Flavonoids | AFI/CRP |
|  |  |  |  | [M-H]^-^ | 609.1825 | 609.1820 | -0.8 | 610.1828, 609.1828, 594.1594, 489.1419, 463.1248, 447.1316, 445.1148, 403.1043, |  |  |
| 43 | Licraside | C_26_H_30_O_13_ | 13.10 | [M+H]^+^ | 551.1759 | 551.1753 | -1.0 | 419.1328, 257.0814, 239.0703, 211.0775, 147.0444, 137.0231, 133.0490, 115.0389, | Flavonoids | GRR |
|  |  |  |  | [M-H]^-^ | 549.1614 | 549.1609 | -0.8 | 549.1622, 417.1189, 297.0772, 255.0651, 135.0075, 119.0498 |  |  |
| 44 | Nomilinic acid glucoside | C_34_H_48_O_16_ | 13.16 | [M+NH_4_]^+^ | 730.3281 | 730.3278 | -0.3 | 730.3306, 533.2363, 429.2273, 427.2089, 395.1884 | Limonoids | AFI/CRP |
|  |  |  |  | [M-H]^-^ | 711.2870 | 711.2864 | -0.7 | 711.2851, 651.2643, 607.2761, 161.0454, 113.0240, 101.0230 |  |  |
| 45 | Glycyroside | C_27_H_30_O_13_ | 13.18 | [M+H]^+^ | 563.1759 | 563.1753 | -1.1 | 563.1675, 269.0814, 213.0892, 177.0556, 145.0285 | Flavonoids | GRR |
|  |  |  |  | [M-H]^-^ | 561.1614 | 561.1610 | -0.6 | - |  |  |
| 46 | Hesperetin 5-O-glucoside | C_22_H_24_O_11_ | 13.19 | [M+H]^+^ | 465.1391 | 465.1388 | -0.8 | 303.0862, 179.0349, 177.0554, 153.0187, 145.0284 | Flavonoids | AFI |
|  |  |  |  | [M-H]^-^ | 463.1246 | 463.1243 | -0.5 | 301.0710, 286.0477, 283.0603, 257.0809, 242.0578, 201.0191, 199.0396, 174.0331, |  |  |
| 47 | Nomilin 17-beta-D-glucopyranoside | C_34_H_46_O_15_ | 13.23 | [M+NH_4_]^+^ | 712.3175 | 712.3172 | -0.4 | 533.2404, 515.2257, 469.2176, 437.1985, 161.0615 | Limonoids | CRP |
|  |  |  |  | [M-H]^-^ | 693.2764 | 693.2761 | -0.4 | 694.2841, 693.2765, 589.2672, 565.2658, 547.2549, 507.2235, 471.1980, 403.2117, |  |  |
| 48 | Isoliquiritin* | C_21_H_22_O_9_ | 13.50 | [M+H]^+^ | 419.1337 | 419.1336 | -0.1 | 257.0813, 242.0563, 239.0702, 211.0745, 163.0397, 147.0444, 137.0230, 123.0448, | Flavonoids | GRR |
|  |  |  |  | [M-H]^-^ | 417.1191 | 417.1190 | -0.2 | 417.1187, 297.0770, 269.0830, 256.0679, 255.0649, 254.0591, 253.0503, 149.0238, |  |  |
| 49 | Ononin* | C_22_H_22_O_9_ | 13.54 | [M+H]^+^ | 431.1337 | 431.1333 | -0.9 | 269.0810, 254.0569, 253.0513, 237.0548, 226.0605, 213.0904 | Flavonoids | GRR |
|  |  |  |  | [M+FA-H]^-^ | 475.1246 | 475.1248 | 0.4 | 268.0697, 267.0652, 266.1673, 252.0434, 44.9978 |  |  |
| 50 | Obacunone glucoside | C_32_H_42_O_13_ | 13.67 | [M+NH_4_]^+^ | 652.2964 | 652.2963 | 0.0 | 456.2055, 455.2058, 411.2158, 409.1989, 161.0614 | Limonoids | AFI |
|  |  |  |  | [M-H]^-^ | 633.2553 | 633.2549 | -0.6 | 633.2541, 589.2625, 427.2146, 359.1834, 331.1895, 287.1994, 161.0463, 119.0351, |  |  |
| 51 | Licorice glycoside C2 | C_36_H_38_O_16_ | 13.69 | [M+H]^+^ | 727.2233 | 727.2232 | -0.1 | 291.0866, 227.0713, 177.0542, 167.0332, 97.0279 | Flavonoids | GRR |
|  |  |  |  | [M-H]^-^ | 725.2087 | 725.2088 | 0.1 | 725.2106, 549.1639, 531.1503, 399.1097, 255.0664, 193.0506, 175.0384 |  |  |
| 52 | Didymin | C_28_H_34_O_14_ | 14.10 | [M+H]^+^ | 595.2021 | 595.2017 | -0.7 | 449.1466, 433.1461, 415.1397, 397.1292, 353.1003, 329.1016, 287.0915, 281.0677, | Flavonoids | AFI |
|  |  |  |  | [M-H]^-^ | 593.1876 | 593.1879 | 0.5 | 593.1871, 351.0903, 309.0783, 285.0772, 241.0922 |  |  |
| 53 | Poncirin | C_28_H_34_O_14_ | 14.28 | [M+H]^+^ | 595.2021 | 595.2019 | -0.5 | 449.1440, 433.1478, 431.1350, 415.1372, 413.1191, 397.1268, 395.1124, 365.1060, | Flavonoids | AFI/CRP |
|  |  |  |  | [M+FA-H]^-^ | 639.1931 | 639.1926 | -0.7 | 594.1907, 593.1865, 473.1424, 447.1309, 431.1349, 429.1186, 387.1076, 369.0975, |  |  |
| 54 | Liquiritigenin* | C_15_H_12_O_4_ | 14.38 | [M+H]^+^ | 257.0808 | 257.0806 | -0.8 | 257.0794, 242.0576, 239.0700, 211.0743, 165.0703, 163.0382, 147.0442, 137.0229, | Flavonoids | GRR |
|  |  |  |  | [M-H]^-^ | 255.0663 | 255.0665 | 0.7 | 255.0662, 135.0084, 119.0495, 117.0346, 93.0338, 91.0189, 41.0032 |  |  |
| 55 | Uralsaponin F | C_44_H_64_O_19_ | 14.48 | [M+H]^+^ | 897.4115 | 897.4109 | -0.6 | 897.4131, 721.3742, 703.3692, 546.3461, 545.3474, 528.3393, 527.3361, 509.3256, | Triterpenoid saponins | GRR |
|  |  |  |  | [M-H]^-^ | 895.3969 | 895.3966 | -0.4 | 896.3972, 895.3926, 773.3767, 351.0577, 193.0336 |  |  |
| 56 | Licoricesaponin A3 | C_48_H_72_O_21_ | 14.49 | [M+H]^+^ | 985.4639 | 985.4633 | -0.6 | 986.4636, 985.4608, 810.4340, 809.4352, 647.3792, 616.3926, 615.3874, 471.3457, | Triterpenoid saponins | GRR |
|  |  |  |  | [M-H]^-^ | 983.4493 | 983.4490 | -0.3 | 984.4529, 983.4482, 821.3950, 803.3854, 351.0559 |  |  |
| 57 | 22-hydroxy-Licorice saponin G2 | C_42_H_62_O_18_ | 14.54 | [M+H]^+^ | 855.4009 | 855.4016 | 0.8 | 643.3382, 503.3330, 486.3278, 485.3271, 141.0188 | Triterpenoid saponins | GRR |
|  |  |  |  | [M-H]^-^ | 853.3863 | 853.3864 | 0.0 | 854.3895, 853.3849, 351.0560, 193.0351, 131.0354 |  |  |
| 58 | Isovitexin 7-O-xylosyl 2''-O-arabinoside | C_31_H_36_O_18_ | 14.55 | [M+H]^+^ | 697.1974 | 697.1974 | 0.0 | 392.1066, 391.1011, 343.0461, 187.0583, 127.0386 | Flavonoids | CRP |
|  |  |  |  | [M-H]^-^ | 695.1829 | 695.1832 | 0.5 | 695.1773, 633.1801, 593.1497, 551.1381, 389.0874, 388.0819, 374.0650, 373.0574, |  |  |
| 59 | Citrusin III | C_36_H_53_N_7_O_9_ | 14.62 | [M+H]^+^ | 728.3978 | 728.3972 | -0.8 | 729.3987, 728.3961, 700.4021, 615.3126, 587.3160, 502.2292, 474.2349, 339.1632, | Peptides | CRP |
|  |  |  |  | [M-H]^-^ | 726.3832 | 726.3835 | 0.4 | 726.3813, 708.3729, 696.3718, 559.3299, 493.3128 |  |  |
| 60 | Bergaptol | C_11_H_6_O_4_ | 14.68 | [M+H]^+^ | 203.0339 | 203.0338 | -0.3 | 203.0341, 159.0441, 147.0456, 131.0488, 119.0476, 91.0547 | Coumarins | AFI/CRP |
|  |  |  |  | [M-H]^-^ | 201.0193 | 201.0193 | -0.4 | 201.0188, 173.0250, 145.0295, 129.0344, 117.0346, 103.0186, 101.0392 |  |  |
| 61 | Natsudaidain 3-(4-O-3-Hydroxy-3-Methylglutaroylglucoside) | C_33_H_40_O_18_ | 14.95 | [M+H]^+^ | 725.2287 | 725.2283 | -0.6 | 726.2297, 725.2264, 420.1374, 419.1342, 404.1110, 389.0863, 127.0387 | Flavonoids | AFI/CRP |
|  |  |  |  | [M-H]^-^ | 723.2142 | 723.2135 | -1.0 | 723.2109, 417.1178, 402.0942, 359.0747, 125.0241, 101.0248, 99.0444, 57.0341 |  |  |
| 62 | 22-Acetoxyglycyrrhizin | C_44_H_64_O_18_ | 14.98 | [M+H]^+^ | 881.4165 | 881.4157 | -1.0 | 706.3921, 705.3891, 530.3557, 529.3528, 512.3463, 511.3422, 451.3243, 405.3159 | Triterpenoid saponins | GRR |
|  |  |  |  | [M-H]^-^ | 879.4020 | 879.4007 | -1.5 | 881.4047, 880.4025, 879.3978, 352.0613, 351.0552, 289.0537, 193.0347, 175.0233, |  |  |
| 63 | 24-Hydroxyl-glycyrrhizin | C_42_H_62_O_17_ | 15.07 | [M+H]^+^ | 839.4060 | 839.4054 | -0.7 | 488.3488, 470.3344, 469.3304, 433.3075, 215.1403 | Triterpenoid saponins | GRR |
|  |  |  |  | [M-H]^-^ | 837.3914 | 837.3911 | -0.4 | 839.4023, 838.3919, 837.3879, 351.0574, 193.0349 |  |  |
| 64 | Naringenin | C_15_H_12_O_5_ | 15.47 | [M+H]^+^ | 273.0757 | 273.0757 | -0.1 | 273.0759, 153.0188, 147.0443, 123.0447, 119.0487, 107.0486, 95.0492, 91.0546 | Flavonoids | AFI/CRP |
|  |  |  |  | [M-H]^-^ | 271.0612 | 271.0610 | -0.9 | 271.0608, 227.0715, 187.0400, 185.0599, 177.0184, 165.0189, 161.0618, 151.0027, |  |  |
| 65 | Citrusin I | C_34_H_53_N_7_O_9_ | 15.59 | [M+H]^+^ | 704.3978 | 704.3976 | -0.3 | 704.3977, 686.3854, 668.3716, 591.3178, 573.3062 | Peptides | CRP |
|  |  |  |  | [M-H]^-^ | 702.3832 | 702.3834 | 0.3 | 614.3320, 596.3292, 503.3008, 447.2348, 410.2383 |  |  |
| 66 | Licorice saponine G2 | C_42_H_62_O_17_ | 15.65 | [M+H]^+^ | 839.4060 | 839.4052 | -0.9 | 840.4121, 839.4067, 664.3718, 663.3702, 646.3658, 645.3597, 627.3518, 488.3448, | Triterpenoid saponins | GRR |
|  |  |  |  | [M-H]^-^ | 837.3914 | 837.3908 | -0.8 | 839.4002, 838.3956, 837.3916, 819.3934, 775.3953, 661.3643, 352.0601, 351.0559, |  |  |
| 67 | Rhaoglycyrrhizin | C_48_H_72_O_20_ | 15.76 | [M-H]^-^ | 967.4544 | 967.4545 | 0.1 | 968.4599, 967.4506, 497.1155, 435.1159, 339.0930 | Triterpenoid saponins | GRR |
| 68 | Licoricesaponin E2 | C_42_H_60_O_16_ | 15.76 | [M+H]^+^ | 821.3954 | 821.3945 | -1.1 | 645.3643, 470.3299, 469.3303, 452.3220, 451.3201 | Triterpenoid saponins | GRR |
|  |  |  |  | [M-H]^-^ | 819.3809 | 819.3802 | -0.8 | 820.3834, 819.3785, 757.3861, 351.0558, 289.0576, 193.0350, 175.0249, 113.0249, |  |  |
| 69 | Homoeriodictyol | C_16_H_14_O_6_ | 15.83 | [M+H]^+^ | 303.0863 | 303.0860 | -0.9 | 303.0845, 179.0350, 177.0542, 153.0175, 149.0591, 145.0284, 137.0599, 134.0369, | Flavonoids | AFI/CRP |
|  |  |  |  | [M-H]^-^ | 301.0718 | 301.0717 | -0.1 | 301.0708, 286.0475, 285.0425, 283.0601, 258.0532, 257.0823, 242.0576, 241.0508, |  |  |
| 70 | Tricin | C_17_H_14_O_7_ | 15.88 | [M-H]^-^ | 329.0667 | 329.0671 | 1.3 | 329.2340, 314.0426, 299.0194, 271.0241, 229.1440, 211.1332, 171.1023 | Flavonoids | BCT |
| 71 | 22β-Acetoxyl licorice saponin C2 | C_44_H_64_O_17_ | 15.91 | [M+H]^+^ | 865.4216 | 865.4210 | -0.7 | 865.5005, 689.3884, 513.3565, 496.3473, 495.3454 | Triterpenoid saponins | GRR |
|  |  |  |  | [M-H]^-^ | 863.4071 | 863.4069 | -0.2 | 864.4100, 863.4055, 352.0598, 351.0575, 193.0349, 175.0249, 113.0238 |  |  |
| 72 | Yunganoside G1 | C_48_H_74_O_21_ | 15.94 | [M+H]^+^ | 987.4795 | 987.4792 | -0.3 | 988.5143, 841.4183, 823.4065, 471.3445, 453.3322 | Triterpenoid saponins | GRR |
|  |  |  |  | [M-H]^-^ | 985.4650 | 985.4649 | -0.1 | 986.4761, 985.4639, 497.1106, 339.0891, 321.0812 |  |  |
| 73 | 5-Hydroxy-3, 6, 7, 8-tetramethoxy-3', 4'-methylenedioxyflavone | C_20_H_18_O_9_ | 15.98 | [M-H]^-^ | 401.0878 | 401.0878 | 0.0 | 401.0883, 357.0594, 313.0720, 269.0790, 225.0544, 121.0288 | Flavonoids | CRP |
| 74 | 7-Hydroxy-5, 6, 8, 3', 4'-Pentamethoxyflavone | C_20_H_20_O_8_ | 16.01 | [M+H]^+^ | 389.1231 | 389.1229 | -0.4 | 389.1231, 374.1000, 359.0748, 341.0674, 316.0592, 197.0071, 169.0144, 163.0749 | Flavonoids | AFI/CRP |
| 75 | Glycyrrhizic acid* | C_42_H_62_O_16_ | 16.11 | [M+H]^+^ | 823.4111 | 823.4100 | -1.2 | 824.4128, 823.4143, 648.3822, 647.3746, 472.3478, 471.3461, 455.3432, 454.3379, | Triterpenoid saponins | GRR |
|  |  |  |  | [M-H]^-^ | 821.3965 | 821.3951 | -1.7 | 822.3979, 821.4035, 818.6225, 803.3881, 759.3976, 645.3644, 627.3511, 352.0600, |  |  |
| 76 | Isoliquiritigenin* | C_15_H_12_O_4_ | 16.29 | [M+H]^+^ | 257.0808 | 257.0806 | -1.1 | 257.0794, 239.0715, 211.0757, 165.0702, 147.0442, 137.0229, 119.0486, 117.0323, | Flavonoids | GRR |
|  |  |  |  | [M-H]^-^ | 255.0663 | 255.0663 | 0.1 | 255.0648, 135.0085, 119.0497, 117.0347, 93.0339, 91.0190, 41.0032 |  |  |
| 77 | 7-Hydroxy-5, 6, 8, 3', 4'-pentamethoxyflavone or its isomer | C_20_H_20_O_8_ | 16.41 | [M+H]^+^ | 389.1231 | 389.1230 | -0.2 | 389.1222, 374.0992, 359.0740, 344.0521, 341.0648, 331.0809, 328.0933, 316.0567, | Flavonoids | AFI/CRP |
|  |  |  |  | [M-H]^-^ | 387.1085 | 387.1085 | 0.0 | 372.0843, 357.0616, 342.0389, 329.0634, 327.0143 |  |  |
| 78 | Formononetin* | C_16_H_12_O_4_ | 16.55 | [M+H]^+^ | 269.0808 | 269.0806 | -0.7 | 269.0813, 254.0588, 253.0499, 237.0535, 226.0623, 225.0541, 213.0921, 198.0666, | Flavonoids | GRR |
|  |  |  |  | [M-H]^-^ | 267.0663 | 267.0663 | 0.1 | 267.0663, 252.0430, 251.0366, 224.0470, 223.0388, 208.0520, 195.0454, 135.0083, |  |  |
| 79 | Licoricesaponine B2 | C_42_H_64_O_15_ | 16.56 | [M+H]^+^ | 809.4318 | 809.4309 | -1.1 | 634.3975, 633.4008, 457.3652, 440.3587, 439.3551 | Triterpenoid saponins | GRR |
|  |  |  |  | [M-H]^-^ | 807.4172 | 807.4165 | -1.0 | 808.4174, 807.4141, 351.0573, 193.0348, 113.0248 |  |  |
| 80 | Licorice saponine H2 | C_42_H_62_O_16_ | 16.63 | [M+H]^+^ | 823.4111 | 823.4109 | -0.2 | 647.3748, 471.3462, 454.3402, 453.3361, 177.1655 | Triterpenoid saponins | GRR |
|  |  |  |  | [M-H]^-^ | 821.3965 | 821.3962 | -0.4 | 822.3981, 821.3949, 351.0559, 193.0351, 175.0250, 113.0249 |  |  |
| 81 | 5, 6, 7, 4'-Tetramethoxyflavone | C_19_H_18_O_6_ | 16.92 | [M+H]^+^ | 343.1176 | 343.1173 | -0.8 | 343.1161, 328.0934, 327.0870, 313.0693, 312.0610, 299.0903, 285.0757, 284.0688, | Flavonoids | AFI/CRP |
| 82 | Uralsaponin A | C_42_H_62_O_16_ | 17.01 | [M+H]^+^ | 823.4111 | 823.4105 | -0.7 | 823.4117, 647.3749, 453.3406, 436.3288, 159.0274 | Triterpenoid saponins | GRR |
|  |  |  |  | [M-H]^-^ | 821.3965 | 821.3967 | 0.2 | 822.4061, 821.3972, 351.0556, 193.0363, 113.0216 |  |  |
| 83 | Limonin* | C_26_H_30_O_8_ | 17.17 | [M+H]^+^ | 471.2013 | 471.2010 | -0.7 | 471.1977, 453.1882, 435.1827, 427.2125, 425.1968, 411.1787, 409.2012, 407.1852, | Limonoids | AFI/CRP |
|  |  |  |  | [M+FA-H]^-^ | 515.1923 | 515.1920 | -0.6 | 469.1882, 411.1461, 381.2037, 349.1472, 323.1651, 321.1126, 306.1281, 305.1525, |  |  |
| 84 | Licorice saponin C2 | C_42_H_62_O_15_ | 17.55 | [M+H]^+^ | 807.4161 | 807.4155 | -0.8 | 807.4049, 631.3873, 438.3467, 437.3412, 419.3243 | Triterpenoid saponins | GRR |
|  |  |  |  | [M-H]^-^ | 805.4016 | 805.4012 | -0.5 | 806.4010, 805.3990, 743.4000, 351.0577, 157.0122 |  |  |
| 85 | 6-Gingerol | C_17_H_26_O_4_ | 17.56 | [M+H]^+^ | 295.1904 | 295.1907 | 1.1 | 147.1175, 137.0596 | Gingerols | PR |
| 86 | Nobiletin* | C_21_H_22_O_8_ | 17.72 | [M+H]^+^ | 403.1387 | 403.1378 | -2.2 | 403.1402, 388.1149, 373.0953, 355.0818, 327.0860 | Flavonoids | AFI/CRP |
| 87 | Oxypeucedanin | C_16_H_14_O_5_ | 17.73 | [M+H]^+^ | 287.0914 | 287.0917 | 1.0 | 287.0913, 161.0601, 153.0174, 118.0399, 105.0690 | Coumarins | AFI/CRP |
|  |  |  |  | [M-H]^-^ | 285.0768 | 285.0769 | 0.1 | 285.0773, 243.0653, 164.0117, 151.0042, 136.0169, 108.0212, 107.0131 |  |  |
| 88 | Glycycoumarin | C_21_H_20_O_6_ | 17.85 | [M+H]^+^ | 369.1333 | 369.1329 | -0.9 | 369.1336, 313.0715, 311.0547, 299.1278, 298.0488, 285.0760, 283.0606, 272.0686, | Flavonoids | GRR |
|  |  |  |  | [M-H]^-^ | 367.1187 | 367.1187 | 0.0 | 367.1168, 352.0942, 351.0862, 337.0707, 309.0386, 297.0397, 293.0813, 284.0324, |  |  |
| 89 | Glyasperin C | C_21_H_24_O_5_ | 18.02 | [M+H]^+^ | 357.1697 | 357.1692 | -1.1 | 357.1688, 301.1072, 221.1178, 191.0692, 179.0704, 165.0545, 153.0566, 149.0603, | Flavonoids | GRR |
|  |  |  |  | [M-H]^-^ | 355.1551 | 355.1548 | -0.8 | 355.1542, 323.1301, 233.1189, 207.1029, 204.0781, 147.0452, 135.0453, 121.0290, |  |  |
| 90 | Sophoraisoflavone A | C_20_H_16_O_6_ | 18.20 | [M+H]^+^ | 353.1020 | 353.1017 | -0.7 | 353.1002, 335.0920, 311.0562, 307.0958, 227.0711, 153.0187 | Flavonoids | GRR/CRP |
|  |  |  |  | [M-H]^-^ | 351.0874 | 351.0873 | -0.4 | 351.0878, 336.0636, 335.0583, 333.0781, 323.0930, 321.0407, 177.0183 |  |  |
| 91 | 3, 5, 6, 7, 8, 3', 4'-Heptamethoxyflavone | C_22_H_24_O_9_ | 18.24 | [M+H]^+^ | 433.1493 | 433.1491 | -0.5 | 433.1488, 418.1245, 417.1195, 403.1012, 400.1160, 399.1105, 388.0773, 387.0710, | Flavonoids | CRP |
| 92 | Licoisoflavone A | C_20_H_18_O_6_ | 18.27 | [M+H]^+^ | 355.1176 | 355.1175 | -0.2 | 299.0899, 287.0553, 229.0859, 161.0601, 123.0443 | Flavonoids | GRR |
| 93 | Licocoumarone | C_20_H_20_O_5_ | 18.44 | [M-H]^-^ | 339.1238 | 339.1237 | -0.2 | 339.1225, 324.0986, 281.0451, 269.0456, 253.0482, 241.0506, 165.0202 | Phenols | GRR |
| 94 | Tangeretin* | C_20_H_20_O_7_ | 18.52 | [M+H]^+^ | 373.1282 | 373.1279 | -0.8 | 373.1293, 358.1045, 343.0832, 325.0711, 297.0757 | Flavonoids | AFI/CRP |
| 95 | Obacunone* | C_26_H_30_O_7_ | 18.61 | [M+H]^+^ | 455.2064 | 455.2063 | -0.2 | 455.2015, 409.2010, 331.1331, 161.0588, 95.0123 | Limonoids | AFI/CRP |
| 96 | Monohydroxy-tetramethoxyflavone | C_19_H_18_O_7_ | 18.91 | [M+H]^+^ | 359.1125 | 359.1124 | -0.3 | 359.1110, 344.0902, 343.0803, 315.0873, 310.0439 | Flavonoids | AFI/CRP |
| 97 | 3-hydroxytangeretin | C_20_H_20_O_8_ | 19.28 | [M+H]^+^ | 389.1231 | 389.1222 | -2.2 | 389.1223, 374.0993, 373.0916, 359.0741, 356.0876, 355.0795, 345.0976, 344.0522, | Flavonoids | AFI/CRP |
| 98 | Poricoic acid B* | C_30_H_44_O_5_ | 20.53 | [M-H]^-^ | 483.3116 | 483.3117 | 0.2 | 483.3088, 411.2866, 410.2777, 409.2740 | Triterpene acids | P |
| 99 | Dehydrotumulosic acid | C_31_H_48_O_4_ | 21.00 | [M+H]^+^ | 485.3625 | 485.3623 | -0.6 | 467.3497, 449.3346, 311.2344, 293.2244, 145.0996 | Triterpene acids | P |
|  |  |  |  | [M-H]^-^ | 483.3480 | 483.3479 | -0.1 | 483.3484 |  |  |
| 100 | Poricoic acid A* | C_31_H_46_O_5_ | 21.13 | [M-H]^-^ | 497.3272 | 497.3273 | 0.0 | 497.3242, 425.3084, 423.2932, 381.3127, 211.1507 | Triterpene acids | P |
| 101 | Tumulosic acid | C_31_H_50_O_4_ | 21.25 | [M+H]^+^ | 487.3782 | 487.3773 | -1.9 | 433.3092, 309.2233, 199.1462, 159.1159, 109.0998 | Triterpene acids | P |
|  |  |  |  | [M-H]^-^ | 485.3636 | 485.3631 | -1.1 | 485.3630 |  |  |
| 102 | Polyporenic acid C | C_31_H_46_O_4_ | 21.86 | [M+H]^+^ | 483.3469 | 483.3461 | -1.6 | 465.3354, 309.2214, 223.1476 | Triterpene acids | P |
|  |  |  |  | [M-H]^-^ | 481.3323 | 481.3325 | 0.3 | 481.3297, 405.2773, 116.9286 |  |  |
| 103 | Pachymic acid* | C_33_H_52_O_5_ | 23.32 | [M-H]^-^ | 527.3742 | 527.3739 | -0.5 | 527.3742 | Triterpene acids | P |

Pinelliae Rhizoma (PR), Aurantii Fructus Immaturus (AFI), Citri Reticulatae Pericarpium (CRP), Glycyrrhizae Radix et Rhizoma (GRR), Bambusae Caulis in Taenias (BCT), Poria (P)
